# Owner-Perception of the Effects of Two Long-Lasting Dog-Appeasing Pheromone Analog Devices on Situational Stress in Dogs

**DOI:** 10.3390/ani12010122

**Published:** 2022-01-05

**Authors:** Céline S. Nicolas, Gemma Espuña, Aurélie Girardin, Jaume Fatjó, Jonathan Bowen, Patricia Monginoux

**Affiliations:** 1 Global Marketing & Market Development Department, Virbac, 13e rue LID, 06511 Carros, France; gemma.espuna@virbac.com; 2 Research & Development Petcare Department, Virbac, 13e rue LID, 06511 Carros, France; aurelie.girardin@virbac.com (A.G.); patricia.monginoux@virbac.com (P.M.); 3 Ethometrix Ltd., 168 Church Road, East Sussex BN3 2DL, UK; jaumefatjo@ethometrix.com (J.F.); jonbowen@ethometrix.com (J.B.)

**Keywords:** appeasing pheromone, dog’s behavior, Zenidog, stress, relaxing, long-lasting collar, behavioral scales, diffusing gel, signs of stress, sources of fear

## Abstract

**Simple Summary:**

Previous studies have found that an analog of the canine-appeasing pheromone can help dogs to relax during stressful situations. Devices diffusing this analog exist but usually last for only one month. Here, two new devices were tested: Zenidog™ collar and Zenidog™ diffusing gel (plug in-free, Virbac, Carros, France), which last for three and two months, respectively. These were compared with existing reference products whose period of effectiveness is one month. The devices were given to owners of dogs showing signs of stress or anxiety in a range of everyday situations. Owners were asked to regularly evaluate and score several parameters that were used to assess changes in a range of behaviors, sources of fear, and specific signs of stress during the three- or two-month studies. A significant improvement in global scores was found for the behaviors and sources of fear and for the stress signs with all the tested devices. There was no difference in effect between the devices. The new collar also seemed effective in puppies and dogs that were wearing an antiparasitic collar. These studies show that the new devices are as effective as the reference products but last longer.

**Abstract:**

Devices that release a synthetic analog of the canine-appeasing pheromone can help to relax dogs during stressful situations, but they usually last for only one month. Two new devices with this analog were tested by owners of dogs showing signs of stress in a range of everyday situations: Zenidog™ collar, lasting three months, and Zenidog™ diffusing gel, lasting two months (Virbac, Carros, France). They were compared against reference products that last for one month. In the three-month study with collars, one group received Zenidog™ collar, one received the reference collar, and one group of dogs wore an antiparasitic collar alongside a Zenidog™ collar. In the two-month study with diffusers, groups received either the unpowered Zenidog™ gel diffuser or the reference electric diffuser. Owners regularly completed a questionnaire that assessed seventeen general behaviors and sources of fear and eleven specific signs of stress. Global scores for these two main scales were calculated, and the evolution of scores was compared between groups. Non-parametric tests with a Bonferroni correction were used for statistical analysis. An improvement of all global scores was observed in all groups (*p* < 0.001), including in puppies, and there was no difference between groups. Zenidog™ devices were as effective as the reference devices and lasted longer.

## 1. Introduction

Anxiety and stress-related problems are amongst the most commonly reported behavior problems in dogs [1]. Chronic stress is usually a major underlying factor in behavior conditions, such as separation-related disorders, noise reactivity, and aggression [1]. Anxiety-related disorders not only have a negative impact on the mental wellbeing of dogs but also on their physical health and lifespan [2]. Furthermore, an increasing range of medical conditions are becoming recognized as being partially caused or aggravated by stress and stress-related traits, such as anxiety, fearfulness, and excitability [3].

Dog appeasing pheromone is a synthetic analog of a pheromone secreted by the intermammary sebaceous glands of the bitch after parturition and has a relaxing effect on puppies [4]. This analog has been extensively studied and has been shown to help reduce signs of stress in different stressful situations even in adult dogs [5,6,7,8,9,10,11,12,13,14,15]. It is available in a range of products including collars and electric diffusers and is used to help relax dogs during stressful events.

These devices generally last for one month, which is shorter than the amount of time needed to manage stress in dogs [5] or to help puppies and young dogs during training and socialization [6]. An effective program of behavioral management would therefore require several refills or replacements of these short-duration devices. Therefore, new alternatives have been developed to provide a longer lasting effect and an eco-friendlier approach, using only one device for several months.

Zenidog™ gel (Virbac, Carros, France) is a diffusing gel that lasts for two months and does not require any power source to operate. Zenidog™ collar (Virbac, Carros, France) is an unpowered collar that diffuses the pheromone analog for three months and is available in two sizes: a small one for puppies and dogs < 10 kg and a larger one for dogs > 10 kg.

In order to test the effectiveness of the new Zenidog™ devices, we conducted two independent studies in dogs showing signs of stress or anxiety according to their owners. In the first study, we compared the new prolonged-release pheromone collar against the reference collar with proven effectiveness but a diffusing period of one month only [5,6,8,9,12,13]. A number of puppies were included in this study to compare the efficacy of the small collars specifically in puppies. A group of dogs wearing an antiparasitic collar was also included in this study since some owners (and veterinarians) may be concerned about using two different collars with the same dog. Although no interaction was expected, the perceived efficiency and tolerance of the tested collar was tested in presence of an antiparasitic collar.

In the second study, we tested the efficacy of the new long-lasting diffusing gel against the reference plug-in product that had shown effectiveness in other studies [11,14,15,16,17,18]. To assess the effectiveness of the devices on behavior and stress at home, two different scales were used by owners: one to assess the general behavior and sources of fear of the dogs, based on 13 behaviors commonly observed by owners and identified in previous studies [19,20,21] and another one assessing specific signs of stress that owners could observe and based on 11 signs, using the commonly used approach of a visual-analog scale [22].

The aim of these studies was to look at the owner’s impression of the effectiveness of the products in the everyday life of the dog, and there was no veterinarian assessment. Owner evaluation of this kind has previously been used in other studies [19,23,24].

## 2. Materials and Methods

### 2.1. Animals and Selection

The study included healthy, owned dogs living in France (and in UK in the study with diffusers) of any breed, age (>3 months old), or sex (but not pregnant or lactating) and not currently being treated for behavioral problems. Dogs were included after screening using a pre-study questionnaire that assessed the general behavior of their animals. Positive health status was a requirement for inclusion, but this was based only on the owner’s report. In the study with collars, a group of 3–6 month-old puppies was specifically recruited in order to assess effectiveness in that age group.

To be included, the dog also had to be single (no other animals in the house), not showing signs of aggression, and:

- Presenting at least one of the following characteristics: fearful, anxious, stressed, hyperactive, distrustful, destructive, solitary (withdrawn), or dirty (eliminating indoor);
- Presenting at least one of the seventeen behaviors or sources of fear listed when replying to the question “Is your dog subject to any of the following behavior?”: licks its paws for no particular reason (no wound, injury, parasites, etc.); barks excessively or when hears a noise; jumps at the slightest noise; low head/neck posture, tail between the legs; growls at people and/or other animals; destroys/shreds objects, furniture, shoes, toys, rubbish bags, etc.; urinates when it is emotionally aroused (excited, anxious, etc.); whines often; often hides or tries to hide; shakes/trembles often; often defecates or urinates in an unusual/inappropriate place; eats its feces; remains inactive all day (immobility, few activities or games); is afraid of thunder and fireworks; does not like to be alone (whines, barks, does not eat…); is afraid during car travel; and is afraid of other animals;
- Or have a mean score of 5 from the list of signs of stress listed and scored when replying to the question: “Stand 2 m away from the dog and observe it for 10 s for the following signs of stress. Please select the signs you detect and rate them from 0 to 10, 10 being the maximum stress for the criterion”: agitation; yawning; paw licking; lip licking; crouched body posture; low tail position; ears back; panting; screaming/barking; hiding/trying to hide; and trembling.

### 2.2. Products and Groups

In the study comparing collars, one group of 45 dogs received the reference collar (Adaptil™ Calm collar S/M or M/L, Ceva, Libourne, France, containing 5% of a synthetic analog of the dog appeasing pheromone, each lasting 1 month), which was changed every month for three months (three in total); one group of 46 dogs received one test collar (Zenidog™ collar S or M/L, Virbac, Carros, France, containing 5% of a synthetic analog of the canine appeasing pheromone, each lasting 3 months) for the 3-month duration of the study; and one group of 37 dogs that were already wearing an antiparasitic collar (AP collar, any brand) received one test collar (Zenidog™ collar, Virbac, Carros, France) for the 3-month duration of the study. The size of the collar was chosen according to the dog’s age or weight and the manufacturer’s instructions.

In the study comparing diffusers, one group of 49 dogs in France and 49 dogs in the UK (98 in total) received the reference diffuser (Adaptil™ Calm electric diffuser, Ceva, Libourne, France, containing 2% of a synthetic analog of the dog appeasing pheromone, which lasted 1 month) refilled each month (two in total), and 47 dogs in France and 54 in UK (101 in total) received one test non-electric diffuser (Zenidog™ diffusing gel, Virbac, Carros, France, containing 6% of a synthetic analog of the canine appeasing pheromone and lasting 2 months) for the 2-month duration of the study.

### 2.3. Procedures

Owners of dogs fulfilling the selection criteria and who agreed to test a calming diffuser or collar received instructions about how to use it. On days 0, 7, 15, 30, 45, and 60 (and 75 and 90 with the collars), the owners completed questionnaires to assess the behavior, sources of fear, and stress signs that they observed in their dogs.

Each time, the owners had to score the presence of the behaviors and sources of fear observed during the screening (as described in “Animals and Selection”), scoring from 0 (no sign) to 10 (very present).

The scale used to assess the dog’s behavior was tailor-made and based on the common signs of stress identified in previous studies that could be easily observed by pet owners on a daily basis [19,20,21]. Signs that could occur in situations that were not related to stress, such as hypersalivation that can occur when the dog is expecting its food or paw lifting that can be used for training or as a game by some owners, and signs that could be recognized only by a veterinarian (e.g., increased respiration and heart rate, muscle rigidity) were excluded from the questionnaire. In the end, the scale included 13 behaviors (including vocalization, destructiveness, and inappropriate elimination) presented in an understandable way by owners and 4 common sources of fear (thunder and fireworks, separation, other animals, car travel) that were likely to happen during the time course of the studies.

The owners also had to score the presence (0: no sign; 10: very present) of the specific signs of stress detected during the screening (described in “Animals and Selection”). This scale was based on the commonly used approach of using a visual-analog scale (VAS), in this instance, including 11 signs of stress [22].

Other questions related to the products’ characteristics and easiness of use, perceived effectiveness, overall impression, and satisfaction with the products were also asked during and at the end of the studies (See Supplementary Materials).

### 2.4. Statistical Analysis

For the analysis of the data, the scores for the seventeen general behaviors and sources of fear were summed to obtain a global score. For the analysis, only the dogs with a global score >3 (with a theoretical maximum of 170) on day 0 were included. Since not all dogs presented the same number of behaviors or fears, the sum was also divided by the number of behaviors or fears reported for each dog.

The same sum (to obtain a global score) and proportion of the number of signs observed per animal were calculated for the eleven signs of stress. Only dogs with a global score >1 (with a theoretical maximum of 110) on day 0 were included in the analysis.

The data analysis was performed using the Real Statistics Resource Pack software (Release 7.6; Copyright (2013–2021) Charles Zaiontz. www.real-statistics.com, last accessed on 6 October 2021).

Due to the nature of the data (ordinal scores) and non-normal distribution of the data (verified with the Shapiro–Wilk test), non-parametric tests were performed. Data are presented as median (first quartile Q1–third quartile Q3) or median (min–max). A Friedman test was used to assess the intra-group evolution of scores during the studies. Since two scales were used to assess the improvement of behaviors and signs of stress, a Bonferroni correction was applied to set the threshold for significance to *p* < 0.025. If a significant difference was observed, comparisons between day 0 and each time point were performed using a pairwise signed-rank test, and the Bonferroni correction for 5 or 7 comparisons (time points of assessment) was applied to set the threshold for statistical significance (*p* < 0.01 or *p* < 0.007 for the studies with the diffusers and the collars, respectively).

To compare groups between them, the percentage of change of the global scores versus day 0 was calculated for each dog and at each time point (global score at day x—global score at day 0)/global score at day 0). Since a decrease of score corresponds to an improvement, the percentage of improvement described in the figures is the positive inverse of the percentage of change (−% change versus day 0). A Kruskal–Wallis test or Mann–Whitney test was performed to compare the percentages of change from day 0 between groups at each time point. The Bonferroni correction for 5 (diffusers) or 7 (collars) comparisons was applied here as well to set the threshold for statistical significance at *p* < 0.01 or *p* < 0.007, respectively. To compare scores given to specific criteria (like product’s approval or perceived effectiveness), a Kruskal–Wallis test (study with collars) or Mann–Whitney test (diffusers) was performed with statistical significance set for *p* < 0.05.

## 3. Results

### 3.1. Characteristics of Selected Dogs

The characteristics of the recruited dogs are described in Table 1. Male and female dogs of any breed and weight were recruited. No preference was given for neutering, but most of the dogs in the collar study had not been neutered. In this latter study, some puppies (three to six months old) were specifically recruited to assess the effect of the small collars on their behavior. The other dogs recruited were mostly above one year old in both studies. No preference was given for the breed and weight of the dogs, but most were between 5 and 30 kg in both studies.

### 3.2. Data on Day 0

On day 0, the most reported of the seventeen behaviors was the dog licking its paws for no particular reason (no wounds or parasites, for example), with 30 to 57% of owners reporting this behavior in the different groups (Table 2). The dog barking excessively or when it hears a noise was the second behavior most reported (38 to 42% of owners, Table 2). Thirty-three to 54% of owners also described their dogs as scared of thunder and fireworks, and 29 to 38% said their dog does not like to be alone. Concerning the signs of stress, the most frequent signs observed were agitation, yawning, paw licking, and lip licking (ranging from 35% to 63% of owners, Table 2).

### 3.3. Evolution of Scores

#### 3.3.1. Study with the Collars

- General behaviors and sources of fear

Taken individually, of the behaviors and sources of fear that were the most represented (*n* ≥ 8 in all groups), those that showed the highest improvement (based on the percentage of decrease of the median score by day 90, see Table S1) were as follows: “licks paws for no particular reasons” (−71%, −50%, and −43% in the groups with the reference collar and the ones with Zenidog™ collar without or with an antiparasitic collar, respectively); “jumps at the slightest noise” (−60%, −18%, and −43%, respectively); “is afraid of thunder or fireworks (−44%, −40%, and −50%, respectively); and “does not like to be alone” (−50%, −17%, and −25%, respectively). Though less represented (*n* = 8, 15, and 3, respectively), the criteria “dog carrying his head or neck low, tail between legs” was the most improved one (−89%, −83%, and −83%, respectively).

The scores given by the owners for each behavior or source of fear were summed to create a global score for the seventeen criteria (general behaviors and fears). Higher scores corresponded with a higher level of stress or anxiety, and changes in score indicated an increase or decrease in stress or anxiety level.

**Figure 1:**
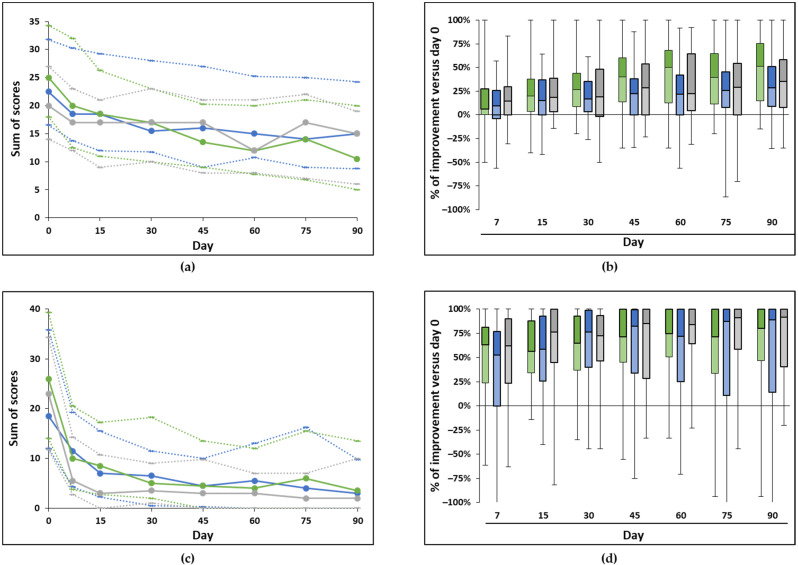
Evolution of the global scores in the study with collars. (**a**,**c**) The global scores for the general behaviors and fears (sum of 17 scores—(**a**)) and the signs of stress (sum of 11 scores—(**c**)) are plotted as median (plain line), first, and third quartiles (dotted lines below and above the median, respectively) for the groups of dogs receiving the reference collar (green) or Zenidog™ collar (blue) and the group of dogs with an antiparasitic collar receiving the Zenidog™ collar (grey). The scores significantly decreased over time (Friedman tests, *p* < 0.0001) in all groups; (**b**,**d**) the percentage of improvement of the global scores versus day 0 (opposite of the % of change) for the general behaviors and fears (**b**) and the signs of stress (**d**) are represented in these box plots (color coding as in (**a**,**c**)). There was no significant difference between groups at any time point (Kruskal–Wallis tests, *p* > 0.05). Number of animals per group as in Table 3.

There was a significant decrease in this global score over time in all groups (*p* < 0.001; Figure 1a, Table 3). Looking specifically at the difference in scores between day 0 and later time points, there was a significant difference as of day seven for the group receiving the reference collar and the group of dogs with an antiparasitic (AP) collar and Zenidog™ collar (*p* < 0.007). For the remaining group (Zenidog™ collar only), the difference fell short of significance on day seven (*p* = 0.01), and the decrease started to be significantly different as of day 15 (*p* < 0.001).

Since the dogs did not show the same numbers of behaviors or sources of fear, the global score obtained per animal was divided by the number of behaviors and sources of fear observed. This ratio significantly decreased from day 0 to day 90 (*p* < 0.001, Table 3), and the pairwise comparison showed similar results as with the global scores.

To compare groups, the percentage of score reduction from day 0 was calculated at each time point assessed and compared between groups. The percentage of improvement (positive inverse of the % of score decrease) at each time point for each group is shown in Figure 1b. No significant difference was found between groups at any time point, showing that both collars (test and reference) and conditions (with or without AP collar) were similar.

Extracting the data for the puppies only (reference collar: *n* = 14; Zenidog™ collar: *n* = 12) also showed a significant improvement over time with both the reference collar and Zenidog™ collar (*p* < 0.01 for both; Figure S1a). There was no significant difference between groups concerning the percentage of score reduction from day 0 at any time point (Figure S1b).

- Signs of stress

All of the eleven signs observed improved by at least 75% by day 90, based on the percentage of decrease of the median scores (Table S1), except for the sign “paw licking,” which decreased by 60% in the reference group. The most improved were as follows: “crouched body posture” (−100% in all groups); “panting” (−100% in all groups); “screaming/barking” (−92%, −100%, and −100% in the groups with the reference collars and the Zenidog™ collar without or with an AP collar, respectively); “hiding/ trying to hide” (−82%, −100%, and −100%, respectively); and “ears back” (−91%, −100%, and −90%, respectively).

Analyzing the scores given for the eleven signs of stress summed up into a global score also showed a significant improvement over time in all groups (*p* < 0.001; Figure 1c, Table 3). The improvement was significant as of day seven in all groups (*p* < 0.001). The analysis of the ratio (global score/number of signs observed) gave similar results (significant decrease as of day seven in all groups, Table 3).

Comparing groups between them by looking at the percentage of score decrease from day 0 showed no statistically significant difference between groups at any time point (Figure 1d).

Extracting the data for the puppies only (Reference collar: *n* = 13; Zenidog™ collar: *n* = 14) also showed a significant improvement over time with both the reference collar and Zenidog™ collar (*p* < 0.01 for both, Figure S1) and no difference between groups concerning the percentage of score reduction from day 0 at any time point (Figure S1d).

- Owners’ perception of the products and perceived effectiveness

At the end of the study (and during the study), the owners were asked if they felt their dog’s behavior improved and were asked to give scores to different parameters concerning the products’ characteristics (see Supplementary Materials). They were also asked general questions about their perception of the products’ effectiveness and approval. Most owners felt the improvement in all groups (58%, 59%, and 65% in the groups with the reference collar, the Zenidog™ collar and the Zenidog™ collar with an AP collar, respectively) and would recommend the collars (64%, 68%, and 75%, respectively, Table S2). The approval scores were similar in all groups (median (Q1–Q3): 7 (5–9), 7 (5–8), and 7 (4–9) in the respective groups; *p* >0.05), and so were the efficacy scores given (7 (5–9), 6.5 (5–8), and 6 (4–8), respectively; *p* > 0.05). Most owners (> 50%) felt their dog was calmer at home, during walks, when travelling, when left alone, or during household activities (Table S2). No differences were observed between groups concerning the different characteristics of the collars (odor, aspect, feeling, overall impression, easiness to handle, etc. Table S3).

Of note, all owners of dogs wearing an antiparasitic collar (*n* = 37) reported no impact (or they did not know) of the pheromone collar on the antiparasitic collar apparent effectiveness or tolerance except for one owner, who saw parasites. This owner also replied he/she changed the AP collar without following instructions.

#### 3.3.2. Study with Diffusers

Similar analysis were performed with the diffusers though the study lasted 60 days, and only two groups were assessed (one with the reference electric diffuser and one with the tested diffusing gel).

- General behaviors and sources of fear

A quick analysis of the scores given for each general behavior or source of fear showed that, among the most represented ones (*n* > 25 in both groups), those that improved the most (based on the percentage of decrease of the median score by day 60, Table S1) were the following: “licks paws for no particular reasons” (−50% and −57% in the groups with the reference diffuser and Zenidog™ diffuser, respectively); “jumps at the slightest noise” (−38% in both groups); “barks excessively” (−44% and −13%, respectively); “fear of thunder or fireworks (−44% and −20%, respectively); and “does not like to be alone” (−38% and −25%, respectively).

**Figure 2:**
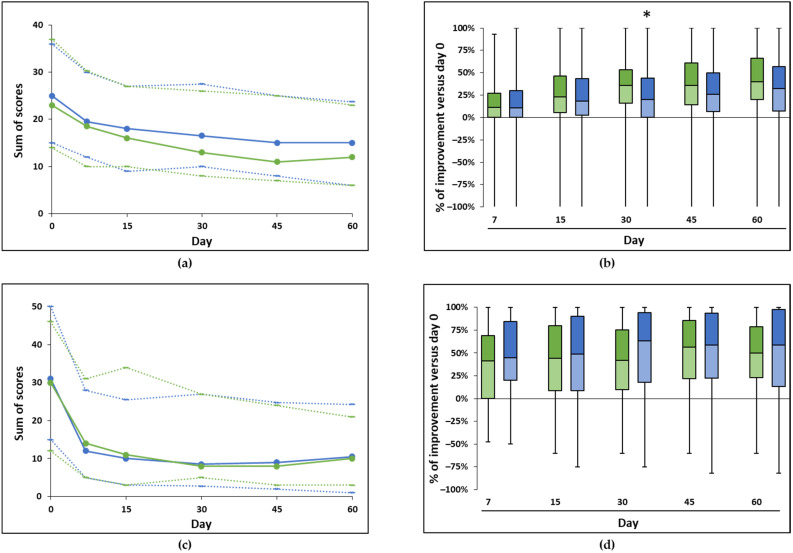
Evolution of scores in the study with the diffusers. (**a**,**c**) The global scores for the general behaviors and fears (sum of 17 scores—(**a**)) and the signs of stress (sum of 11 criteria—(**c**)) are plotted as median (plain line), first, and third quartiles (dotted lines below and above the median, respectively) for the groups of dogs receiving the reference diffuser (green) or Zenidog™ diffuser (blue). The sum of scores significantly decreased over time (Friedman tests, *p* < 0.0001) in all groups. (**b**,**d**) The percentage of improvement of the global scores (opposite of the % of variation) versus day 0 for the general behaviors and fears (**b**) and the signs of stress (**d**) are represented in these box plots (color coding as in (**a**,**c**)). There was no significant difference between groups at any time point for both global scores except at day 30 for the behaviors and fears (Mann–Whitney tests, *: *p* < 0.01). Number of animals per group as in Table 4.

The global scores for the seventeen general behaviors and sources of fear significantly decreased over time in both groups (*p* < 0.001, Figure 2a, Table 4). The pairwise comparisons showed a significant score decrease as of day seven in both groups (*p* < 0.001). Dividing the global scores by the number of behaviors and sources of fear reported per animal showed similar results (significant decrease by day seven, *p* < 0.001, Table 4)

Comparing groups between them based on the percentage of score decrease versus day 0 showed no difference between groups at any time point except on day 30, at the advantage of the group with the reference diffuser (*p* < 0.01, Figure 2b, Table 4).

- Signs of stress

All signs of stress improved by at least 20%, and most improved by at least 50%, based on the percentage of decrease of the median score by day 60 (Table S1). Those that improved the most were as follows: “hiding/ trying to hide” (−75% and −100% in the reference and Zenidog™ group, respectively); “ears back” (−57% and −100%, respectively); “low tail position” (−50% and −91%, respectively); “screaming/barking” (−67% and −71%, respectively); and “trembling” (−80% and −45%, respectively).

The global scores assessing the specific signs of stress also significantly decreased over time in both groups (*p* < 0.001, Figure 2c, Table 4), with a significant score decrease as of day seven in both groups (*p* < 0.001, Figure 2c). The evolution of the ratio when dividing by the number of signs observed per dog showed similar results (significant decrease by day seven, *p* < 0.001).

There was no difference between groups when comparing the percentage of change from day 0 at any time point (Figure 2d, Table 4)

- Owners’ perception of the products and perceived effectiveness

Most owners observed an improvement in both groups (62% and 50% in the groups with the reference diffuser and the Zenidog™ diffusing gel, respectively) and would recommend the product (72% and 64%, respectively, Table S2). The approval scores were similar in both groups (median (Q1–Q3): 7 (5–8) and 7 (4–9) in the respective groups; *p* > 0.05; Table S2), and so were the efficacy scores given (7 (5–8) and 6 (3–8), respectively; *p* > 0.05). Most owners put the diffusers in the sitting/dining room, with an average size mostly ranging between 10 and 40 m² though some owners put it in larger rooms (Table S4). Most owners felt their dog was calmer at home with the diffusers (Table S2). No differences were observed between groups concerning the characteristics of the diffusers (odor and easiness of use, Table S4)

These owner’s impressions confirm the similar effectiveness between the electric diffuser and the longer-lasting diffusing gel to improve the dog’s behaviors at home.

## 4. Discussion

In these studies, the effectiveness of a long-lasting collar and long-lasting plug-in free diffusing gel was compared to devices lasting one month since only these short-lasting devices were available on the market. Furthermore, the plug-in free diffusing gel had to be compared to an electric diffuser since, again, no other plug-in free diffusing gel with the dog-appeasing pheromone analog was available.

The reference collar and diffuser have shown effectiveness in different stressful situations, including during separation from the owner [14], loud noises [8], car travel [5], and when introducing a puppy to its new home [13]. Most studies involved the evaluation of specific criteria by a specialized veterinarian. It is, indeed, not always easy for owners to detect the stress signs in their dogs, some of them being very subtle and not necessarily recognized as a marker of stress [21]. In our studies, we wanted to get the owner’s impressions of the effect of the pheromone analog diffusing devices on their dog’s general behavior in their natural environment and when guided to look at specific signs of stress.

We asked them to score seventeen behaviors or sources of fear generally observed in anxious dogs and that could also be assessed by owners [20,21]. They included active behaviors, such as jumping when hears a noise, barking excessively, or destroying objects; repetitive behaviors, such as licking the paws or lips for no reason; inhibition behaviors, such as hiding or remaining prostrated; or autonomic behaviors, such as urinating or defecating in unusual places or when emotionally aroused, whining, panting, or trembling. Four common sources of fear were also included, such as the fear of thunder and fireworks, the fear of car travel, the fear of other animals, and the fear of human separation. The behavior that was the most observed was the dog licking its paws for no reason (no wound or parasites for example). This parameter can be seen as a sign of overgrooming or excessive licking. Though not specific, it is often seen in dogs with anxiety or stress-related disorders [20,21]. This behavior was also one of those that showed the largest improvement in both studies, together with the dog carrying its head low and tail between its legs and with the dog jumping at the slightest noise or barking excessively.

Unsurprisingly, a significant number of dogs (33% to 54% per group) showed a fear of thunder or fireworks, a very common fear in dogs [1]. This fear seemed to be decreased with the use of the appeasing pheromone, as described previously [8,15]. The global scores for the behaviors and sources of fear decreased significantly in all groups and in all studies with no difference between groups, suggesting that all collars and diffusers tested were effective in calming the dogs. This finding also suggests that all devices are equally efficient in reducing the dog’s responses to stress at home.

These results were further corroborated with the improvement of the signs of stress. Indeed, the owners were asked to score very specific signs of stress when observing their dogs for a few seconds. They had to look at the dog’s body posture and reaction (crouched posture, low tail position, ears back, trembling, panting) and at their behavior (agitation, yawning, licking, barking, hiding). Agitation, yawning, and licking (paws or lips) were the most cited in the study with collars, while ears back was more cited in the study with the diffusers. The signs that improved the most concerned the body posture and the excessive reactions, such as barking or hiding. The global scores for the signs of stress greatly improved on day seven by more than 40% in all groups and all studies to reach at least 80% of improvement at the end of the studies, while the improvement for the general behaviors and sources of fear was lower (maximum of 51% of improvement at the end).

Indeed, scoring very specific signs of stress during a certain time seems to be a more objective way to evaluate reactions to stress than when evaluating general behaviors. When evaluating the latter, several parameters can skew the scoring, including how the owner is perceiving this behavior and how it impacts its quality of life. It is also more subject to interpretation, with terms like “often” or “excessively” in the questionnaire. Finally, the fact that the difference in improvement between groups tended to be greater when evaluating the general behaviors and sources of fear than when evaluating the signs of stress is also in favor of a difference in objectivity. Indeed, it has to be noted that the reference products used in these studies, which have been on the market for quite some time, were well known and advertised, while the tested products were new. This difference may have influenced the owner in perceiving an improvement in the general behaviors or sources of fear with the reference products. However, when assessing more objective signs of stress, such an apparent difference between the reference and tested products was not observed. The difference in reputation between products could also explain the slight (but not significant) difference in the perceived effectiveness of the products by owners.

The rapid onset of action observed (significant differences in scores as of day seven—not assessed at earlier time point) is typical of the pheromone mode of action, which is via specific receptors in the vomeronasal organ that are connected with the amygdala and other parts of the limbic system [4]. This observation is consistent with other studies in which a behavioral improvement has been seen in a few days [8,11,12,13].

Finally, in the study with collars, we also wanted to test if the new pheromone collar (Zenidog™) was effective in puppies and in dogs wearing an antiparasitic collar. The results showed that the Zenidog™ collar was as effective as the reference collar in puppies and that wearing an antiparasitic collar did not seem to impact the effectiveness or tolerance of the Zenidog™ collar, based on the owner’s assessment. This was to be expected since the mechanisms of action are completely different between the pheromones and the active ingredients of antiparasitic collars. However, this result may reassure owners and prescribers who still hesitate to put two different collars on the same dog.

It must be accepted that the evaluation of behaviors by owners is a major limitation of this study. Veterinarians and behaviorists are more experienced in detecting signs of stress in dogs. Many studies have focused on assessments of pheromone’s efficiency during a specific triggering event by expert veterinarians, and this remains the gold standard. However, such an approach does have its own limitations, including very specific criteria and situations and assessment of only one source of stress [6,8,12,13,14]. Our objective was to look at a broader assessment of everyday stress, fear, and anxiety that is more reflective of the dog’s wellbeing, which can only be evaluated by the owner and has been effective in other studies [19,23]. Although the owners reported on their dog’s behavior at pre-set times, it is reasonable to expect that the stress signs and responses to sources of fear were assessed during a specific stressful event that occurred during the test interval and not an event that occurred specifically at the pre-set time. An absence of the source of fear between two pre-set times could also explain, in part, the lack of effect observed by some owners. Another limitation is the fact that the behaviors and signs of stress to observe were unspecific, and some may be the results of dermatological (e.g., licking paws), neurological (e.g., trembling), or systemic diseases (e.g., inactivity). Though only healthy animals, according to their owners, were selected, such underlying diseases cannot be excluded since no veterinarian assessment was involved. These underlying diseases could explain the lack of efficacy of the pheromone product perceived by some owners. However, since, in general, the efficacy of the product was perceived, it can be surmised that most signs observed were indeed of behavioral origin. The placebo effect in this type of non-medical product is also not negligible, especially since the owners knew they were testing a calming diffuser or collar that may have influenced their perception. In this study, no dogs were given a placebo. However, comparing a new product with one that has been shown to be effective in multiple previous studies (including those with a placebo control group [6,8,12,13,14]) is an ethical way to construct an efficacy study that minimizes the use of animals and does not expose any of them to a non-treatment group.

## 5. Conclusions

In conclusion, all the tested devices performed similarly and were able to relax the dogs and reduce the signs of stress, as assessed by the owners at home. No significant difference in effectiveness was observed between the reference devices and the newly developed devices (Zenidog™) at the end of the studies. Furthermore, the effectiveness of the Zenidog™ collar did not seem to be impacted by a concomitant antiparasitic collar worn by the dogs. The main differences in products tested concerned the duration of pheromone release and presentation. The reference collar and electric diffuser can diffuse for one month, while the tested collar and plug in-free diffusing gel can last for three and two months, respectively. A longer duration (meaning less products required per year) with no electricity required could allow an appropriate behavioral management in a more ecological way.

## Figures and Tables

**Table 1:** Characteristics of the dogs in the two different studies. The number of dogs who completed the studies and the percentage of dogs in each category per group are shown in this table. AP, antiparasitic.

	Study with Collars			Study with Diffusers	
	Reference Collar	Zenidog™ Collar	Zenidog™ + AP Collars	Reference Diffuser	Zenidog™ Diffusing Gel
Total number of dogs	45	46	37	98	101
Sex					
Male	60%	54%	65%	45%	47%
Female	40%	46%	35%	55%	53%
Neutered/spayed					
Yes	31%	20%	32%	57%	60%
No	69%	80%	68%	43%	40%
Age					
Between 3 and 6 months	31%	30%	0%	1%	1%
Between 6 and 11 months	0%	2%	5%	3%	2%
1 to 3 years old	22%	24%	38%	18%	28%
4 to 6 years old	20%	13%	24%	30%	21%
7 to 9 years old	9%	13%	24%	26%	25%
More than 10 years old	18%	17%	8%	21%	22%
Body weight					
<5 kg	13%	17%	8%	17%	16%
5 to 10 kg	40%	37%	27%	35%	35%
10 to 30 kg	40%	39%	46%	39%	42%
30 to 50 kg	7%	7%	19%	8%	5%
>50 kg	0%	0%	0%	0%	1%

**Table 2:** Percentage of owners reporting the presence of the specific behaviors, sources of fear, and signs of stress on day 0, sorted by decreasing incidence per category. The number of dogs recruited and the number of behaviors, sources of fear, or signs of stress reported per animal (median (min–max)) is also presented.

	Study with Collars			Study with Diffusers	
	Reference Collar	Zenidog™ Collar	Zenidog™+ AP Collars	Reference Diffuser	Zenidog™ Diffusing Gel
Total number of dogs recruited	45	46	37	98	101
Behaviors and sources of fear reported on day 0 (% of dogs)					
Licks paws for no particular reason	44%	57%	30%	38%	45%
Barks excessively or when hears a noise	38%	39%	41%	42%	38%
Jumps at the slightest noise	22%	17%	24%	28%	32%
Growls at people and or other animals	20%	11%	27%	23%	27%
Carries the head and/or the neck low, tail between the legs	18%	33%	8%	12%	13%
Urinates when emotionally aroused	24%	17%	11%	18%	15%
Whines often	11%	11%	22%	18%	23%
Destroys/shreds objects, furniture, shoes, toys, garbage cans, etc.	18%	22%	16%	13%	9%
Shakes/trembles often	11%	17%	5%	16%	20%
Often hides or tries to hide	16%	20%	3%	7%	12%
Often defecates or urinates in an unusual/inappropriate place	11%	11%	11%	9%	9%
Eats its feces	13%	7%	8%	8%	4%
Remains inactive all day	7%	0%	3%	2%	5%
Afraid of thunder and fireworks	33%	43%	46%	54%	49%
Does not like to be alone	38%	33%	32%	29%	29%
Afraid during car travels	22%	7%	5%	16%	13%
Afraid of other animals	9%	7%	11%	15%	17%
Number of behaviors and fears reported per dog (median; min–max)	3 (0–8)	3 (1–8)	3 (0–6)	3 (1–12)	3 (1–14)
Specific signs of stress reported on day 0 (% of dogs)					
Agitation	56%	61%	59%	36%	38%
Yawning	58%	63%	43%	37%	41%
Paw licking	51%	57%	46%	35%	45%
Lip licking	51%	52%	43%	36%	38%
Ears back	44%	46%	27%	38%	46%
Panting	36%	48%	32%	43%	36%
Low tail position	42%	39%	41%	35%	36%
Crouched body posture	42%	41%	41%	24%	35%
Screaming/barking	44%	33%	35%	32%	27%
Trembling	36%	22%	32%	32%	30%
Hiding/trying to hide	27%	30%	43%	19%	22%
Number of stress signs reported per dog (median; min–max)	4 (1–11)	4 (1–11)	3 (1–11)	2 (0–11)	3 (0–11)

**Table 3:** Change in global scores over time in the study with the pheromone collars. The global scores for behaviors and sources of fear and for the signs of stress are given on day 0 and day 90. The global scores divided by the number of general behaviors and sources of fear or signs of stress noticed per animal (global score/nb) are also given. The percentage of change of the scores from day 0 to the end of the study (day 90) are reported as the “% change”. Data are presented as median (Q1; Q3). * depicts a statistically significant difference between day 0 and day 90 (*p* < 0.007; signed-ranked tests with Bonferroni’s correction for multiple comparisons). No difference was observed in the percentage of change between groups (*p* > 0.05, Kruskal–Wallis tests). AP, antiparasitic.

	Reference Collar		Zenidog™ Collar		Zenidog™ + AP Collars	
	Day 0	Day 90	Day 0	Day 90	Day 0	Day 90
General behaviors and fears						
n	44	44	44	44	33	33
Global score	25	10.5	22.5	15	20	15
	(18; 34.25)	(5; 20) *	(16.5; 31.75)	(8.75; 24.25) *	(14; 27)	(6; 19) *
Global score/nb	7	3.3	7	4.5	6.8	4.3
	(5.9; 8)	(1.7; 5.3) *	(5.8; 8.5)	(3.4; 6.5) *	(5.5; 7.3)	(2.7; 5.5) *
% change		−51%		−29%		−35%
		(−75%; −15%)		(−51%; −9%)		(−58%; −8%)
Specific signs of stress						
n	44	44	46	46	36	36
Global score	26	3.5	18.5	3	23	2
	(14; 39.25)	(0; 13.5) *	(12; 35.75)	(0; 9.75) *	(11.75; 34.25)	(0; 10) *
Global score/nb	5.6	1.1	5.2	0.8	5.8	0.5
	(5.1; 6.7)	(0; 3.1) *	(5; 6)	(0; 4.1) *	(5.3; 6.7)	(0; 2.8) *
% change		−80%		−89%		−91%
		(−100%; −47%)		(−100%; −14%)		(−100%; −40%)

**Table 4:** Evolution of the global scores in the study with the pheromone diffusers. The global scores for the general behaviors and sources of fear and for the specific signs of stress are given on day 0 and day 60. The global scores divided by the number of general behaviors and sources of fear or signs of stress noticed per animal (global score/nb) are also given. The percentage of change of the scores from day 0 are reported as the “% change.” Data are presented as median (Q1; Q3). * depicts a statistically significant difference between day 0 and day 60 (*p* < 0.01; signed-ranked tests with Bonferroni’s correction for multiple comparisons). No statistically significant difference was observed in the percentage of change between groups (*p* > 0.05, Mann–Whitney tests).

	Reference Electric Diffuser		Zenidog™ Diffusing Gel	
	Day 0	Day 60	Day 0	Day 60
General behaviors and fears				
*n*	44	44	44	44
Global score	25 (18; 34.25)	10.5 (5; 20) *	22.5 (16.5; 31.75)	15 (8.75; 24.25) *
Global score/nb	7 (5.9; 8)	3.3 (1.7; 5.3) *	7 (5.8; 8.5)	4.5 (3.4; 6.5) *
% change		−51% (−75%; −15%)		−29% (−51%; −9%)
Specific signs of stress				
*n*	44	44	46	46
Global score	26 (14; 39.25)	3.5 (0; 13.5) *	18.5 (12; 35.75)	3 (0; 9.75) *
Global score/nb	5.6 (5.1; 6.7)	1.1 (0; 3.1) *	5.2 (5; 6)	0.8 (0; 4.1) *
% change		−80% (−100%; −47%)		−89% (−100%; −14%)

## Data Availability

The data presented in this study are available upon request from the corresponding author.

## Supplementary Materials

The following are available online at https://www.mdpi.com/article/10.3390/ani12010122/s1, Supplementary methods; Table S1: Percentage of score reduction for each behavior, source of fear, or sign of stress; Table S2: Satisfaction with products and perceived effectiveness in all groups; Table S3: Summary of replies to specific questions in the study with collars; Table S4: Summary of replies to specific questions in the study with diffusers; Figure S1: Evolution of the global scores in puppies in the study with collars.
