# Supplementary material for: Owner-Perception of the Effects of Two Long-Lasting Dog-Appeasing Pheromone Analog Devices on Situational Stress in Dogs
Source: Animals (Basel). 2022 Jan 5;12(1):122. doi: 10.3390/ani12010122 (PMC8749783; doi:10.3390/ani12010122)
Supplement: Supplementary file 1 [file animals-12-00122-s001.zip › Supplementary methods.pdf]

Owner-Perception of the Effects of Two Long-Lasting Dog-Appeasing Pheromone Analog Devices on Situational Stress in Dogs

Supplementary methods:

Other questions asked to the owners for the trial with the collars included:

- on Day 0
  - On a scale from 0 to 10, can you tell us about the visual aspect (color, material, etc.) of the collar? (0: not attractive at all - 10: very attractive)
  - On a scale of 0 to 10, when you took the collar in hand, how did it feel? (0: very unpleasant - 10: very pleasant)
  - On a scale of 0 to 10, rate your overall impression of the collar? (0: Very bad - 10: excellent)
- on Day 7 and regularly
  - If you had to remove the collar, how easy was it to remove it (0: not easy at all - 10: very easy)
  - If you had to remove the collar, how easy was it to put it back in place (0: not easy at all - 10: very easy)
  - During the period, did the collar come loose or did it fall off (Yes / No)
  - Did you have to readjust the length of the collar during the study to accommodate your pet's growth? (Yes / No)
  - If yes, On a scale of 0 to 10, rate the easiness of adjustment of the collar (0: not easy at all - 10: very easy)
  - On a scale of 0 to 10, rate the visual appearance (color, material, etc.) of the collar today (0: not attractive at all - 10: very attractive)
  - What is your dog's behavior towards the collar? (He doesn't pay attention / He seems to show signs of discomfort)
  - If signs of discomfort, please check the signs below (scratching / shaking his head / rubs against wall /whines / other:specify) and indicate their intensity /frequency (Light or occasional, moderate or regularly; intense or very often)
  - GLOBALLY, since you put the collar on your dog, would you say his behavioral problems were: Greatly improved /Improved/ Unchanged / Degraded / Strongly degraded
  - OVERALL, what liking rating do you give this collar? (0-I didn't like it; 10: I liked it very much)
  - How would you rate the effectiveness of the product concerning your dog's well-being (reduced stress, fears, distrust…)? (0- very ineffective - 10: very effective)
- On Day 90
  - On a scale of 0 to 10, rate your overall impression of the collar (0: Very bad - 10: Excellent)
  - During the test, did you feel your animal was more peaceful than usual:
    - at home (yes /no / not applicable)
    - during walks (yes /no / not applicable)
    - with other dogs (yes /no / not applicable)
    - With other animals (dogs excluded) (yes /no / not applicable)
    - In unusual or new situations (yes /no / not applicable)
    - During visits to the veterinarian (yes /no / not applicable)
    - When he is alone (yes /no / not applicable)
    - When kept by another person (yes /no / not applicable)

    - During thunderstorms, fireworks, firecrackers ... (yes /no / not applicable)
    - During your household activities (vacuum cleaner, washing machine ...) (yes /no / not applicable)
    - When a new member arrives in the family (partner, baby, etc.) (yes /no / not applicable)
- Would you recommend the tested collar to other dog owners? (Yes certainly, Yes probably, Probably not, Certainly not)
- If you noticed an odor when opening the sachet:
    - can you rate the smell perceived when opening the sachet (0: Very unpleasant - 10: very pleasant)
    - Was the smell noticeable when your dog was wearing the collar? (Yes for the duration of the test / Yes, during the first few days / No)
- For dogs with an antiparasitic collar on day 30 and day 60:
- Since putting on the collar to be tested, do you have the feeling that the antiparasitic collar is: (as effective / less effective / I do not know)
- if less effective, what makes you say so? (I saw fleas, ticks, lice.../ He scratches more / other: give details)
- During the test, did you renew the antiparasitic collar according to the duration of use instructions? (Yes, I renewed it following the instructions; No, I renewed it without following the instructions; I didn't need to renew it)
- Do you have the impression that your dog does not tolerate the antiparasitic collar as well since the start of the test? (Yes: specify /No)

Other questions asked to the owners for the trial with the diffuser included:

- on Day 0 or before
- Where is your dog spending most of its time?
- What is the size of this room? (< 10 m²/ 10-20 m²/ 20-30 m²/ 30-40 m²/ 40-50 m²/ 50-60 m²/ 60-70 m²/ >70 m²)
- on Day 1 and regularly
- Would you say that the product is easy to use? (Yes absolutely / yes somewhat / not really / not at all)
- In which room did you install the diffuser? (In the sitting room or dining room / In the bedroom where you sleep/ Another room dedicated to your dog (office, storage room, etc.)/ In the kitchen / In the bathroom / Other)
- Have you noticed any change of smell in the room since the diffuser was used? (Yes / No)
- If yes, can you indicate the intensity of this smell? (0: Discreet / 10: strong) and how do you find this smell? (0: unpleasant/ 10: pleasant)
- on day 7 and regularly
- How does your dog react with regard to the diffuser? (doesn't pay attention/ slightly interested (rubbing, circling, sniffing) / moderately interested (rubbing, circling, sniffing) / very interested in it (rubbing, circling, sniffing))
- OVERALL, since you installed this diffuser, the dog's behavioural problems have: Improved significantly/ Improved / Not changed / Deteriorated / Deteriorated significantly
- OVERALL, what LIKING rating do you give this collar? (0-I didn't like it; 10: I liked it very much)
- How would you rate the effectiveness of the product concerning your dog's well-being (reduced stress, fears, distrust…)? (0- very ineffective - 10: very effective)
- At the end of the study:
- During the test, did you feel your animal was more peaceful than usual:
    - At home (yes /no / not applicable)

    - During walks (yes /no / not applicable)
    - With other dogs (yes /no / not applicable)
    - With other animals (dogs excluded) (yes /no / not applicable)
    - In unusual or new situations (yes /no / not applicable)
    - During visits to the veterinarian (yes /no / not applicable)
    - When he is alone (yes /no / not applicable)
    - When kept by another person (yes /no / not applicable)
    - During thunderstorms, fireworks, firecrackers ... (yes /no / not applicable)
    - During your household activities (vacuum cleaner, washing machine ...) (yes /no / not applicable)
    - When a new member arrives in the family (spouse, baby, etc.) (yes /no / not applicable)
- Would you recommend the diffuser to other dog owners? (Yes certainly, Yes probably, Probably not, Certainly not)
