# Supplementary material for: Owner-Perception of the Effects of Two Long-Lasting Dog-Appeasing Pheromone Analog Devices on Situational Stress in Dogs
Source: Animals (Basel). 2022 Jan 5;12(1):122. doi: 10.3390/ani12010122 (PMC8749783; doi:10.3390/ani12010122)
Supplement: Supplementary file 1 [file animals-12-00122-s001.zip › Supplementary tables.pdf]

Owner-Perception of the Effects of Two Long-Lasting Dog-Appeasing Pheromone Analog Devices on Situational Stress in Dogs

Supplementary Tables

Table S1 : Percentage of score reduction for each behavior, source of fear or sign of stress. The number of animals showing the characteristic is indicated in brackets. In bold are the most improved criteria among the most represented ones (n≥8 in each group in the study with collar and n≥ 25 in the study with diffusers).

	**Study with pheromone collars**			**Study with pheromone diffusers**	
	**Reference collar**	**Zenidog collar**	**Zenidog + AP collars**	**Reference diffuser (electric)**	**Zenidog diffusing gel**
**% of score decrease by the end of the study (behaviors and sources of fear)**					
**Licks paws for no particular reason**	**-71% (n=20)**	**-50% (n=26)**	**-43% (n=11)**	**-50% (n=37)**	**-57% (n=45)**
**Barks excessively or when hears a noise**	-14% (n=17)	-20% (n=18)	0% (n=15)	**-44% (n=41)**	**-13% (n=38)**
**Jumps at the slightest noise**	**-60% (n=10)**	**-18% (n=8)**	**-43% (n=9)**	**-38% (n=27)**	**-38% (n= 32)**
Growls at people and / or other animals	-14% (n=9)	-29% (n=5)	-17% (n=10)	-29% (n=23)	-40% (n=26)
Carries the head and / or the neck low, tail between the legs	-89% (n=8)	-83% (n=15)	-83% (n=3)	-20% (n=12)	-83% (n=13)
Urinates when emotionally aroused	-75% (n=11)	-35% (n=8)	-36% (n=4)	-43% (n=17)	-20% (n=14)
Whines often	-50% (n=5)	0% (n=5)	-45% (n=8)	-38% (n=18)	-38% (n=23)
Destroys / shreds objects, furniture, shoes, etc.	-56% (n=8)	-10% (n=10)	-43% (n=6)	-57% (n=13)	-29% (n=8)
Shakes/trembles a lot	-63% (n=5)	-50% (n=8)	0% (n=2)	-43% (n=15)	-64% (n=20)
Often hides or tries to hide	-83% (n=7)	-29% (n=9)	-29% (n=1)	-57% (n=7)	-38% (n=11)
Often urinates or defecates in an unusual / inappropriate place	-14% (n=5)	-57% (n=5)	- 83% (n=4)	-33% (n=9)	-63% (n=9)

Eats his feces	-27% (n=6)	-89% (n=3)	-100% (n=3)	-47% (n=8)	-80% (n=4)
Remains inactive all day (immobility, few activities or games)	-83% (n=3)	N/A (n=0)	-33% (n=1)	-27% (n=2)	-44% (n=4)
**Afraid of thunder and fireworks**	**-44% (n=15)**	**-40% (n=20)**	**-50% (n=17)**	**-44% (n=53)**	**-20% (n=49)**
**Does not like to be alone**	**-50% (n=17)**	**-17% (n=15)**	**-25% (n=12)**	**-38% (n=28)**	**-25% (n=29)**
Afraid of car travels	-50% (n=10)	0% (n=3)	-50% (n=2)	-50% (n=16)	-57% (n=13)
Afraid of other animals	-60% (n=4)	0% (n=3)	-20% (n=4)	-29% (n=15)	-38% (n=17)
**% of score decrease by the end of the study (signs of stress)**					
Agitation	-86% (n=25)	-100% (n=28)	-91% (n=22)	-50% (n=35)	-50% (n=38)
Yawning	-83% (n=26)	-80% (n=29)	-80% (n=16)	-20% (n=36)	-60% (n=41)
Paw licking	-60% (n=23)	-90% (n=26)	-83% (n=17)	-57% (n=34)	-50% (n=45)
Lip licking	-75% (n=23)	-92% (n=24)	-100% (n=16)	-33% (n=35)	-50% (n=38)
**Ears back**	**-91% (n=20)**	**-100% (n=21)**	**-90% (n=10)**	**-57% (n=37)**	**-100% (n=46)**
**Panting**	**-100% (n=16)**	**-100% (n=22)**	**-100% (n=12)**	-80% (n=42)	-40% (n=36)
**Low tail position**	-80% (n=19)	-100% (n=18)	-100% (n=15)	**-50% (n=34)**	**-92% (n=36)**
**Crouched body posture**	**-100% (n=19)**	**-100% (n=19)**	**-100% (n=15)**	-55% (n=24)	-67% (n=35)
**Screaming/barking**	**-92% (n=20)**	**-100% (n=15)**	**-100% (n=13)**	**-67% (n=31)**	**-71% (n=27)**
**Trembling**	-77% (n=16)	-100% (n=10)	-100% (n=12)	**-80% (n=31)**	**-45% (n=30)**
**Hiding/trying to hide**	**-82% (n=12)**	**-100% (n=14)**	**-100% (n=16)**	-75% (n=19)	-100% (n=22)

Table S2: Satisfaction with products and perceived effectiveness in all groups. Data are presented as median scores (Q1 - Q3) with the number of respondents indicated in italic (white cells) or as percentage of respondents replying “yes” to the question (grey cells). No difference between groups was observed in any study.

	**Study with pheromone collars**			**Study with pheromone diffusers**	
	**Reference collar**	**Zenidog collar**	**Zenidog + AP collars**	**Reference diffuser (electric)**	**Zenidog diffusing gel**
Liking/approval rating at end of study	7 (5 - 9)<br>*45*	7 (5 -8)<br>*46*	7 (4 - 9)<br>*37*	7 (5 -8)<br>*98*	7 (4 - 9)<br>*101*
Effectiveness on dog’s well-being at end of study	7 (5 - 9)<br>*45*	6.5 (5 -8)<br>*46*	6 (4 - 8)<br>*37*	7 (5 -8)<br>*98*	6 (3 - 8)<br>*101*
% of owners perceiving an improvement of behavior	58%	59%	65%	62%	50%
Recommendation rate	64%	68%	75%	72%	64%
During the test, did you feel your pet was calmer than usual? (% yes)					
At home	69%	65%	73%	67%	58%
On a walk	56%	60%	60%	32%	34%
With other dogs	49%	57%	42%	32%	34%
With other animals (excluding dogs)	44%	49%	35%	17%	19%
In unusual or new situations	58%	43%	47%	32%	24%
When travelling in the car	62%	56%	49%	33%	35%
During visits to the vet	54%	44%	35%	14%	22%
When it is alone	60%	53%	48%	54%	44%
When it is looked after by another person	48%	55%	48%	19%	19%
During storms, fireworks, firecrackers, etc.	45%	42%	35%	35%	31%
During household activities (vacuum cleaner, washing machine, etc.)	50%	50%	56%	48%	40%
When a new member arrives in the family (partner, baby, etc.)	50%	56%	35%	24%	16%

Table S3: Summary of replies to specific questions in the study with collars. Data are presented as median scores (Q1 - Q3) with the number of respondents indicated in italic (white cells) or as percentage of respondents replying “yes” to the question (grey cells).

	**Reference collar**	**Zenidog collar**	**Zenidog + AP collars**
Odor when opening (% yes)	29%	15%	11%
Odor perception / approval	5 (3 - 6)<br>*13*	5 (3.5 - 5)<br>*7*	5.5 (4 -6.75)<br>*4*
Odor still present on the dog (% of yes over those who perceived an odor)	77% (10/13)	71% (5/7)	75% (3/4)
Visual aspect (color, material, etc.)	7 (5 - 9)<br>*45*	7 (6 -8.75)<br>*46*	7 (5 - 9)<br>*37*
Collar feel in hand	8 (6 - 9)<br>*45*	8 (6.25 - 9)<br>*46*	7 (6 -8)<br>*37*
Overall first impression	7 (6 - 9)<br>*45*	8 (7 - 9)<br>*46*	7 (6 -8)<br>*37*
Easiness to remove the collar	7.5 (5 - 9)<br>*18*	9 (8 - 10)<br>*24*	8 (7.25 -8.75)<br>*26*
Did the collar come loose or did it fall off? (% yes)	7%	15%	17%
Easiness to put the collar back in place	7 (4 - 8)<br>*41*	9 (7 - 10)<br>*73*	8 (6 - 8)<br>*71*
Did you have to readjust the length of the collar during the study? (% yes)	15%	11%	11%
Easiness of collar adjustment	8 (7.25 - 9)<br>*46*	8 (7 - 9)<br>*36*	8 (5 - 9)<br>*29*
Visual appearance on the dog (evaluated several times)	8 (6 - 8)<br>*315*	7 (6 - 8)<br>*322*	7 (6 - 8)<br>*259*
Dog’s reaction to device (% of dogs showing signs of discomfort)	4%<br>(mostly moderate)	12%<br>(mostly light)	3%<br>(mostly light)

Table S4: Summary of replies to specific questions in the study with diffusers. Data are presented as median scores (Q1 - Q3) and the number of respondents indicated in italic (white cells) or as percentage of respondents (grey cells).

	**Reference diffuser**	**Zenidog diffusing gel**
Size of room where the diffuser was placed (% of respondents - n=80 in each group)		
< 10 m²	4%	4%
10 - 20 m²	30%	19%
20 - 30 m²	34%	31%
30 - 40 m²	21%	24%
40 - 50 m²	5%	15%
50 - 60 m²	1%	4%
60 - 70 m²	3%	1%
> 70 m²	3%	3%
Change of odor in the room (evaluated at all time points) (% yes)	13%<br>(75/587)	9%<br>(54/605)
Odor intensity	4 (3 - 7)<br>*75*	5 (2 - 7)<br>*54*
Odor liking / approval	5 (5 - 7)<br>*75*	5 (3 - 6.75)<br>*54*
Easiness of use (% yes)	100%	100%
