# Supplementary material for: Owner-Perception of the Effects of Two Long-Lasting Dog-Appeasing Pheromone Analog Devices on Situational Stress in Dogs
Source: Animals (Basel). 2022 Jan 5;12(1):122. doi: 10.3390/ani12010122 (PMC8749783; doi:10.3390/ani12010122)
Supplement: Supplementary file 1 [file animals-12-00122-s001.zip › Figure S1 article Zenidog HUT - rev.pdf]

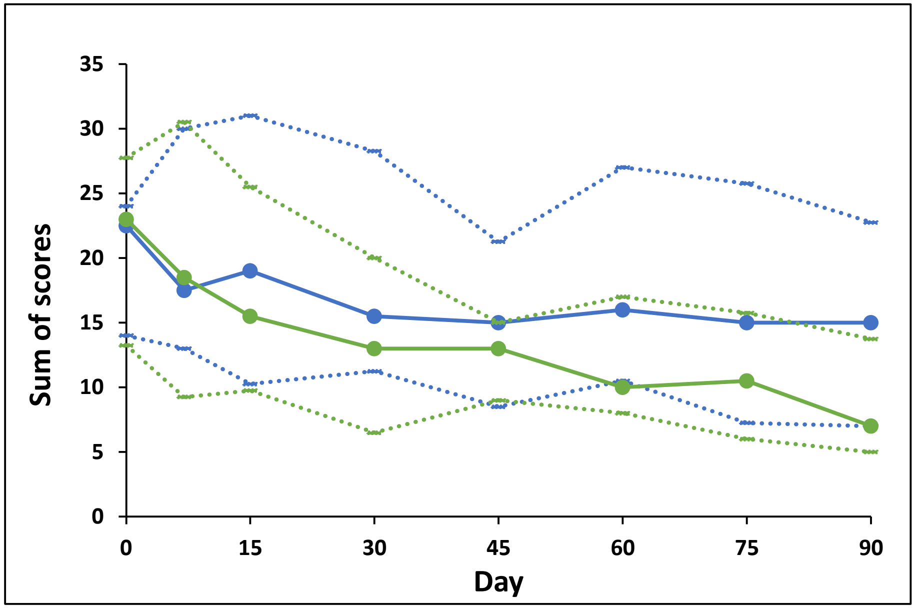

Sum of scores
35
30
25
20
15
10
5
0
0
15
30
45
60
75
90
Day

(a)

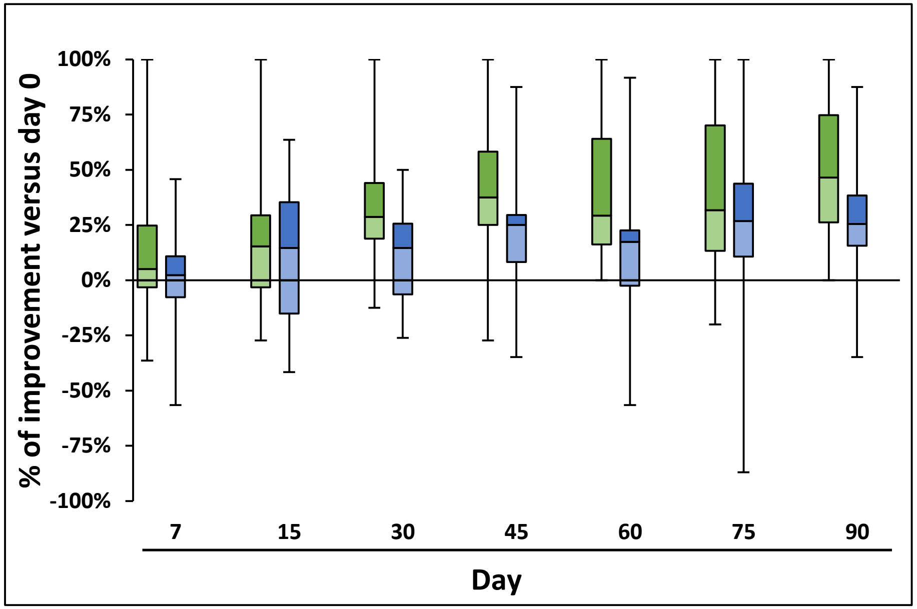

% of improvement versus day 0
100%
75%
50%
25%
0%
-25%
-50%
-75%
-100%
7
15
30
45
60
75
90
Day

(b)

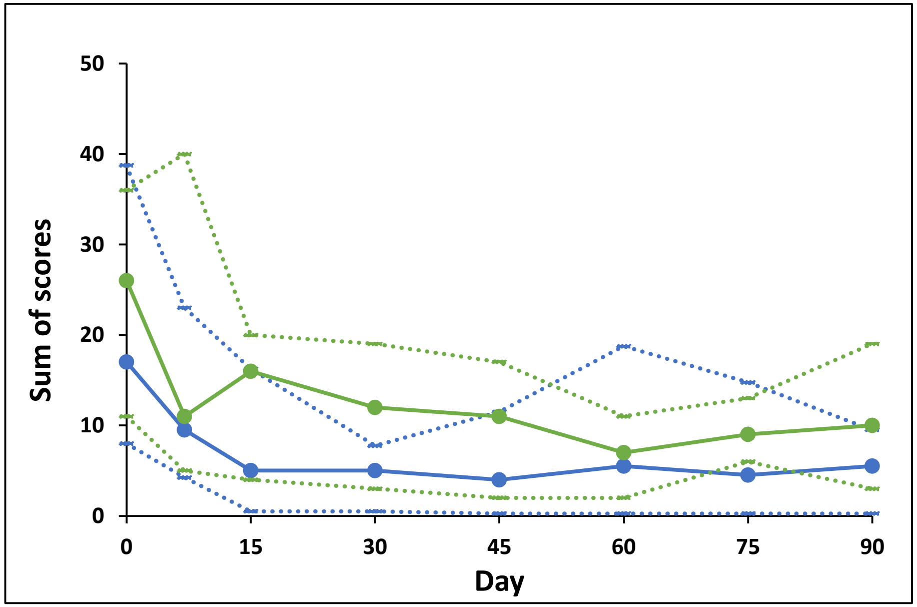

Sum of scores
50
40
30
20
10
0
0
15
30
45
60
75
90
Day

(c)

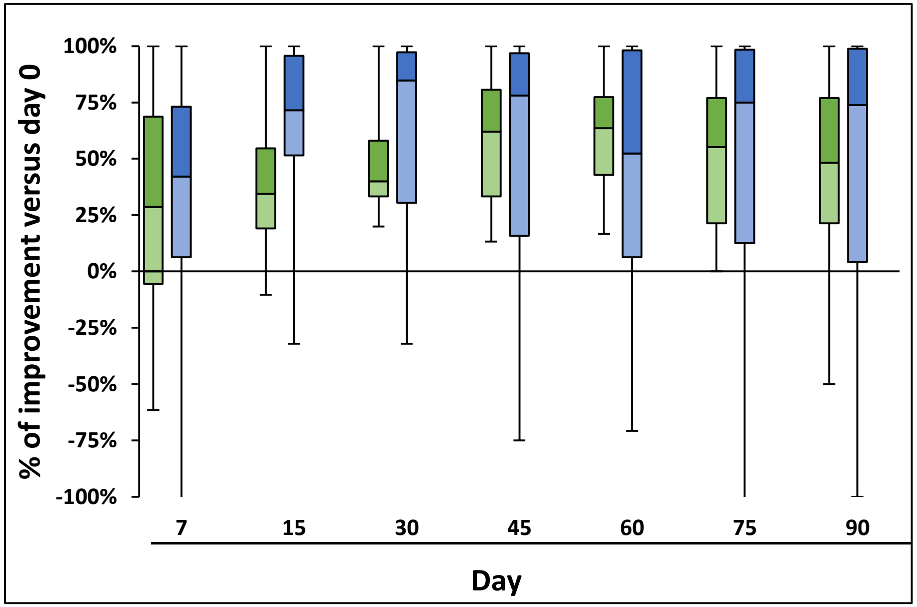

% of improvement versus day 0
100%
75%
50%
25%
0%
-25%
-50%
-75%
-100%
7
15
30
45
60
75
90
Day

(d)

Figure S1. Evolution of the global scores in puppies in the study with collars: (**a**,**c**) The global scores for the general behaviors and fears (sum of 17 scores - **a**) and the signs of stress (sum of 11 scores - **c**) are plotted as median (plain line), first and third quartiles (dotted lines below and above the median, respectively) for the groups of dogs receiving the reference collar (green) or Zenidog collar (blue). The scores significantly decreased over time (Friedman tests, p<0,01) in all groups (reference collar: n=14; Zenidog collar: n=12). (**b**,**d**) The percentage of improvement of the global scores (opposite of the % of variation) versus day 0 for the general behaviors and fears (**b**) and the signs of stress (**d**) are represented in these box plots (color coding as in a and c). There was no significant difference between groups at any time point (Kruskall-Wallis tests, p>0,05, reference collar: n=13; Zenidog collar: n=14).
